# Three-dimensional soft tissue changes according to skeletal changes after mandibular setback surgery by using cone-beam computed tomography and a structured light scanner

**DOI:** 10.1186/s40510-019-0282-0

**Published:** 2019-07-01

**Authors:** Kyung-A Kim, Ye-Jin Chang, Su-Hyun Lee, Hyun-Joon An, Ki-Ho Park

**Affiliations:** 1 Department of Orthodontics, School of Dentistry, Kyung Hee University, 1 Hoegi-Dong, Dongdaemoon-Ku, Seoul, 130-701 South Korea; 2 Department of Orthodontics, Graduate School, Kyung Hee University, Seoul, South Korea

**Keywords:** 3D soft tissue change, Mandibular setback surgery, Class III, CBCT, Facial scan image, Structured light scanner

## Abstract

**Background:**

To evaluate the three-dimensional (3D) changes after mandibular setback surgery (MSS) in skeletal Class III malocclusion using cone-beam computed tomography (CBCT) and a structured light-based scanner.

**Methods:**

Twenty-eight adult Korean patients with skeletal Class III malocclusion treated by MSS were evaluated. CBCT and facial scan images were recorded one week before and six months after surgery. To use an identical 3D coordinate system, superimposition was performed, and nine skeletal and 18 soft tissue landmarks were identified. Changes in the landmarks and correlation coefficients and ratios between hard and soft tissue changes were evaluated. Paired *t* test and Pearson’s correlation test were performed.

**Results:**

After MSS, the amount of transverse correction was 2.45 mm; mandibular setback, 5.80 mm; and vertical reduction, 1.64 mm at the menton, on average. In the transverse axis, there were significant changes and correlations in the lips and chin and an increasing gradient of ratios from the lower lip to the chin. In the anteroposterior axis, the lower lip and chin moved backward significantly and showed notable correlation with hard tissue movement. In the vertical axis, significant upward movement was observed in the landmarks related to the chin, but only lower facial height was significantly decreased.

**Conclusions:**

Soft tissue changes according to hard tissue movement after MSS exhibited a distinct pattern of an increasing gradient from the lips to the chin in a transverse aspect.

## Background

Patients with severe mandibular prognathism resulting in a Class III malocclusion have been treated with a combined orthodontic and orthognathic surgical procedures [1]. Treatment planning for patients with mandibular prognathism should not only correct the malocclusion involving the stomatognathic function but also consider facial esthetic. Esthetic problems associated with malocclusion often cause social handicap and psychological disorders. It is therefore important for the clinician to be able to analyze and predict soft tissue changes [2, 3].

However, there are only a few studies of 3D soft tissue changes using facial scan imaging after orthognathic surgery in mandibular prognathism [4–6]. Although the soft tissue changes were analyzed using 3D values, the hard tissue changes were measured using 2D values with a lateral cephalogram. In previous studies using 2D lateral cephalograms, skeletal changes after orthognathic surgery were evaluated only in an anteroposterior or vertical dimension, but could not be assessed in a transverse dimension from a frontal aspect [4–6].

Cone-beam computed tomography (CBCT) provides 3D information about deep skeletal structures and superficial skin but has disadvantages in soft tissue evaluation due to a low resolution with large slice gaps, the absence of color values, and a long scanning time [7, 8]. Using a structured light scanning system, texture and color information of the face can be readily obtained in high resolution without additional radiation hazards, together with some advantages such as a short scan time, no hazard to the naked eyes, and easy operability [9]. Therefore, CBCT and surface scanning data should be combined to evaluate the relationship between hard and soft tissue changes more accurately.

Therefore, the purpose of this study was to evaluate the 3D changes and correlation between movements of hard and soft tissue after mandibular setback surgery (MSS) in patients with skeletal Class III malocclusion using CBCT and a structured light-based scanner.

## Methods

### Subjects

This retrospective study was approved by the Institutional Review Board of Kyung Hee University Medical Center (IRB No: K-2013-11018794). Skeletal Class III patients who had received bilateral sagittal split ramus osteotomy only by the same surgeon at the Kyung Hee University Dental Hospital had been screened. The subjects were evaluated based on the inclusion and exclusion criteria. Exclusion criteria were as following: (1) severe facial asymmetry (menton deviation > 4 mm), (2) severe transverse discrepancies (more than 4 mm), (3) craniofacial anomaly, (4) subjects with a body mass index greater than 30 kg/m^2^, and (5) subjects with increased or decreased body weight more than 5 kg before and after surgery. A pilot study and power analysis showed that a sample size of at least 24 patients was needed for a 20% effect size change to represent a statistically significant difference. The sample size was calculated a power of 0.80 at a significance level *α* of 0.05. Consequently, 28 adult Korean subjects (13 men and 15 women; mean age, 24.15 ± 4.25 years) were included in this study. The subjects were characterized by normal positioned maxilla (SNA 80.46 ± 2.68°) and prognathic mandible (SNB 82.37 ± 2.41°).

### Data acquisition

CBCT and facial scans were taken one week before and six months after surgery (T0 and T1, respectively). The CBCT image data was obtained with a PSR-9000N® dental CT system (Asahi Roentgen Ind. Co., Ltd., Kyoto, Japan; 80 kV, 5.0 mA, 17-s scan time, 0.3 × 0.3 × 0.3 mm voxel size, and 153.6 × 153.6 mm field of view). Facial scans were performed from three different views simultaneously, using a white-structured light scanner (Morpheus 3D Neo; Morpheus, Gyoung-gi, Korea) (scan time 0.8 mm field of view s, 3 × frame rate 15 frames/s) with the lips relaxed in a natural head position. Three scanning images were reconstructed into one 3D image using a registration process [10].

### Landmarks and coordinate system

**Fig. 1:**
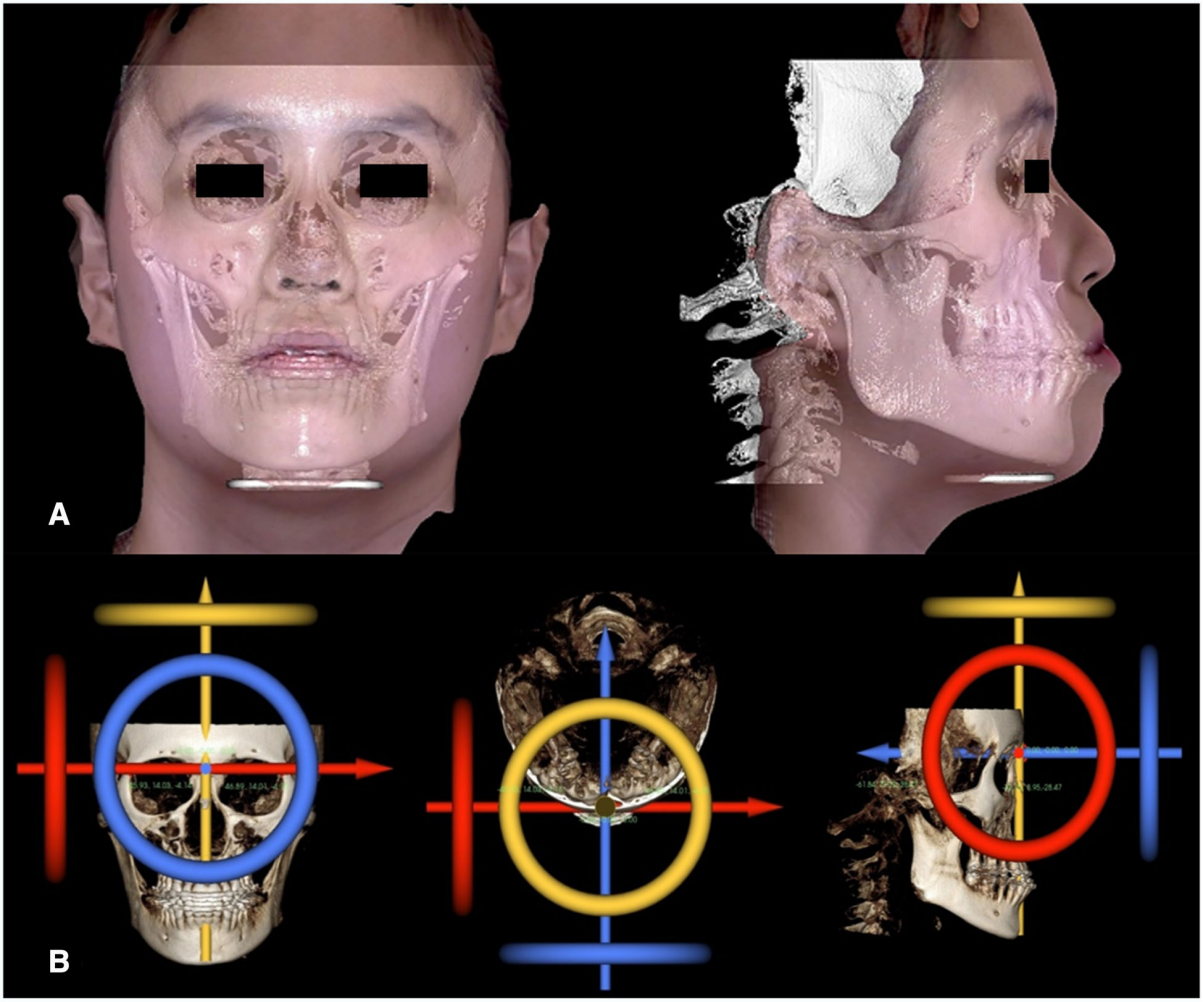
Superimposed 3D image of CBCT and facial scan (**a**) and 3-dimensional coordinate system (**b**)

A 3D coordinate system was established in CBCT images as follows: the transverse axis (*x*), parallel to the frontozygomatic suture point (FZ) line; the anteroposterior axis (*y*), perpendicular to the FZ line and parallel to the right Frankfort horizontal (FH) line; and the vertical axis (*z*), perpendicular to *x*- and *y*-axes. Then, the origin point (0, 0, 0) was set at the nasion point (*N*). The facial scan image was registered on the skin image of CBCT in order to use the same 3D coordinate system of the CBCT image (Fig. 1). Two images were automatically registered by voxelization, skin segmentation, and the Chamfer distance transformation using the Morpheus 3D software [11].

**Fig. 2:**
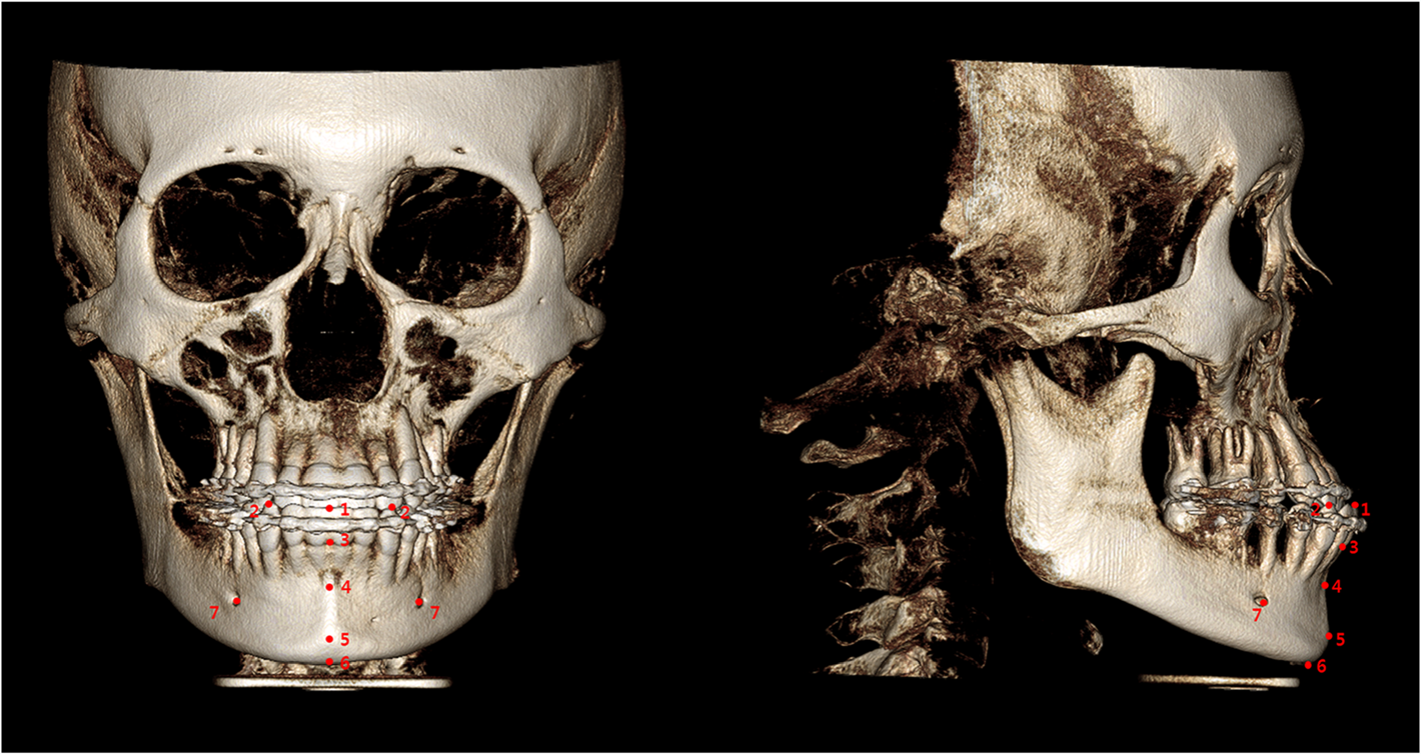
Skeletal landmarks on CBCT images: 1, incision inferior (Ii); 2, lower canine (Lc) on deviated side (dev) and contralateral side (ctl); 3, infradentale (Id); 4, B point (B); 5, pogonion (Pog); 6, menton (Me); and 7, mental foramen (Mf dev/ctl). See also Table 1 for the description of 3D CBCT landmarks

**Fig. 3:**
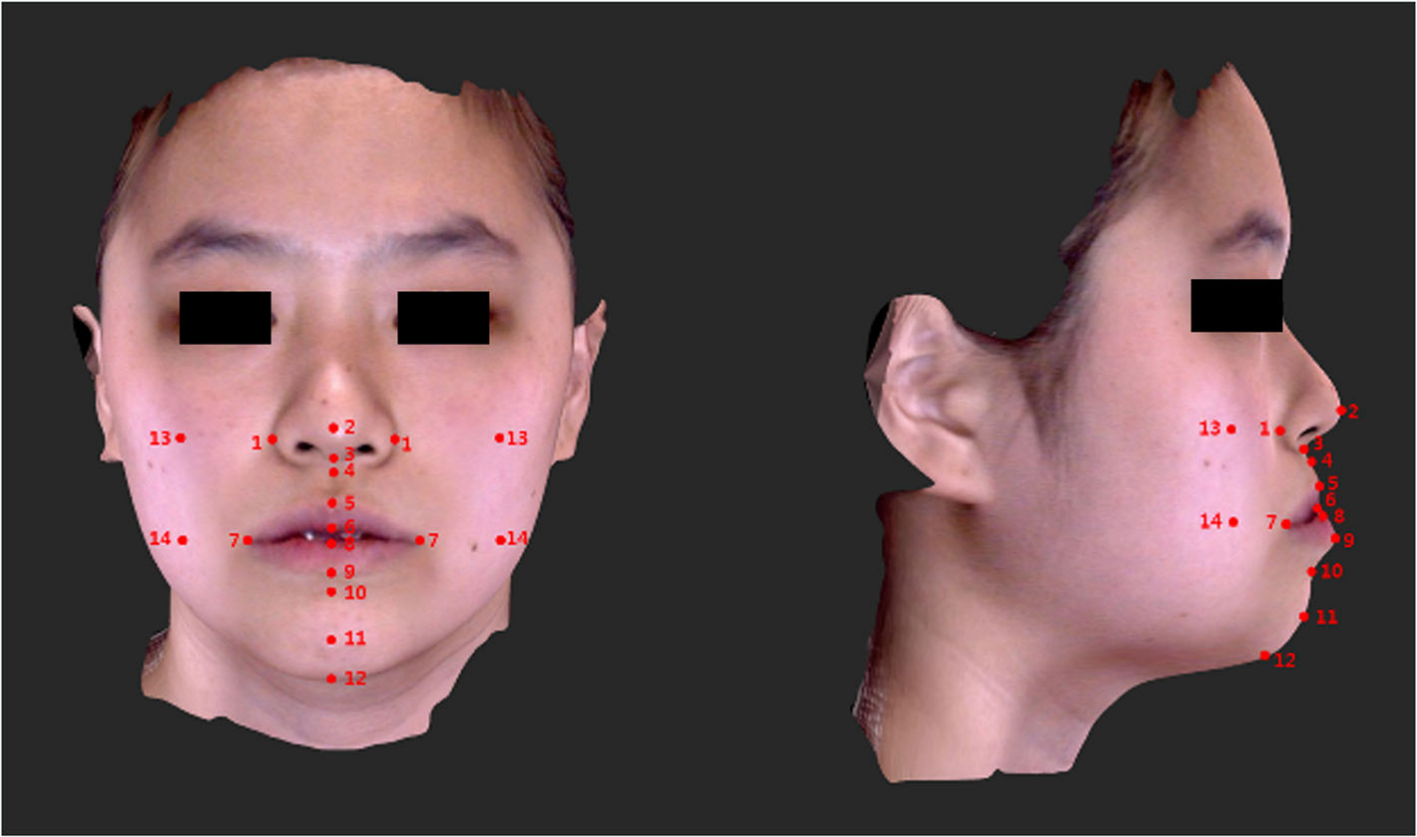
Soft tissue landmarks on facial scan images. 1, nasal ala (Al dev/ctl); 2, pronasale (Pn); 3, subnasale (Sn); 4, A’ point; 5, labrale superius (Ls); 6, stomion superius (Smts); 7, cheilion (Ch dev/ctl); 8, stomion inferius (Stmi); 9, labrale inferius (Li); 10, B’ point (B′); 11, pogonion’ (Pog’); 12, menton’ (Me’); 13, zygomatic point (Zy dev/ctl); and 14, cheek point (Ck dev/ctl). See also Table 2 for the description of 3D soft tissue landmarks

**Fig. 4:**
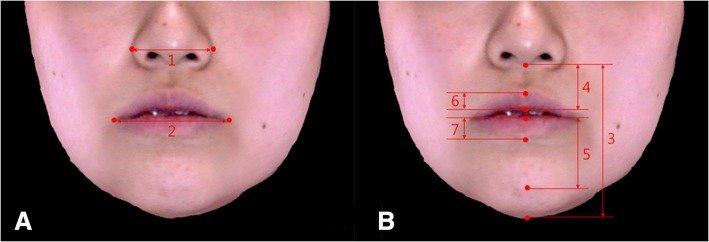
Soft tissue linear measurements. **a** 1. Nasal width (Al dev–Al ctl), 2. Lip width (Ch dev–Ch ctl). **b** 3. Lower facial height (Sn–Me’), 4. Upper lip height (Sn–Stms), 5. Lower lip height (Stmi–Pog’), 6. Upper vermilion height (Ls–Stms), and 7. Lower vermilion height (Li–stmi). See also Table 3 for the description of soft tissue linear measurements

A total of nine skeletal and 18 soft tissue landmarks were identified (Figs. 2 and 3, Tables 1 and 2) and measured in the 3D coordinate values (*x*, *y*, *z*) before and after surgery. A positive (+) sign indicated the menton-deviated side and anterior and superior side to *N* of the subject. A negative (−) sign indicated the opposite. The changes in the landmarks, correlation coefficients (*p*), and soft-to-hard tissue movement ratios (S/H, soft tissue movement/hard tissue movement) were evaluated in transverse, anteroposterior, and vertical axes. In addition, seven linear measurements of soft tissue were evaluated (Fig. 4, Table 3). Digitization and measurement were performed by one investigator.

**Table 1:** Description of three-dimensional (3D) cone-beam CT (CBCT) landmarks in the study

Landmarks	Definition
Incision inferior (Ii)	Midpoint of incisal edge of the mandibular central incisors
Lower canine (Lc dev/ctl)	The highest point of mandibular canine cusp tip on deviated side (dev)/contralateral side (ctl)
Infradentale (Id)	The most anterior point on the apex of the alveolar process between the mandibular central incisors
B point (B)	The deepest point in the anterior outline of the mandible between infradentale and pogonion in the sagittal plane
Pogonion (Pog)	The most anterior point in the mandibular chin area in the sagittal plane
Menton (Me)	The most inferior point in the middle of the mandibular chin in the frontal plane
Mental foramen (Mf dev/ctl)	The most inferior point on the lower edge of mental foramen on deviated side/contralateral side

**Table 2:** Description of 3D soft tissue landmarks in the study

Landmarks	Definition
Exocanthion (Ex)	The outer corner of the eye fissure where the eyelids meet
Endocanthion (En)	The inner corner of the eye fissure where the eyelids meet
Nasal ala (Al dev/ctl)	The most lateral point on each alar contour on deviated side/contralateral side
Pronasale (Pn)	The most protruded point of the apex nasi
Subnasale (Sn)	The midpoint of the angle at the columella base where the lower border of the nasal septum and the surface of the upper lip met
A’ point (A’)	The deepest point in the soft tissue contour of the upper lip
Labrale superius (Ls)	The midpoint of the upper vermilion line
Stomion superius (Stms)	The lowest point of upper lip vermilion
Cheilion (Ch dev/ctl)	The point located at each labial commissure on deviated side/contralateral side
Stomion inferius (Stmi)	The highest point of lower lip vermilion
Labrale inferius (Li)	The midpoint of the lower vermilion line
B’ point (B’)	The most deepest point from lateral view, on the facial midline, between the lower lip and chin
Pogonion’ (Pog’)	The most anterior midpoint of chin
Menton’ (Me’)	The lowest median landmark on the lower border of the mandible
Zygomatic point (Zy dev/ctl)	The point where a vertical line from exocanthion and a horizontal line from nasal ala meet on deviated side/contralateral side
Cheek point (Ck dev/ctl)	The point where a vertical line from exocanthion and a horizontal line from cheilion meet on deviated side/contralateral side

**Table 3:** Description of soft tissue linear measurements in the study

Landmarks	Definition
Nasal width (Al dev–Al ctl)	Distance between nasal ala of the deviated side and contralateral side
Lip width (Ch dev–Ch ctl)	Distance between cheilion of the deviated side and contralateral side
Lower Facial height (Sn–Me’)	Distance between subnasale and menton’
Upper lip height (Sn–Stms)	Distance between subnasale and stomion superius
Lower lip height (Stmi–Pog’)	Distance between stomion inferius and pogonion’
Upper vermilion height (Ls–Stms)	Distance between labrale superius and stomion superius
Lower vermilion height (Li–stmi)	Distance between labrale inferius and stomion inferius

### Statistical analysis

All measurements were repeated after two weeks, and a paired *t* test revealed no difference between the two assessments (*p* > 0.05). Therefore, the second set of measurements was used. After confirming normality of the data distribution using the Shapiro–Wilk test, paired *t* test was performed to compare the values between T1 and T2. Pearson’s correlation test was conducted to assess the degree of correlations between hard tissue change and soft tissue change after orthognathic surgery. Also, soft-to-hard tissue movement ratios were defined. The level of significance was set at *p* < 0.05.

## Results

### Changes in hard tissue landmarks

There were significant changes in most skeletal landmarks in transverse, anteroposterior, and vertical axes (Table 4). The average amount of transverse correction was 2.41 mm at B (*p* < 0.001) and 2.49 mm at Pog (*p* < 0.01); mandibular setback, 5.80 mm at B and 5.69 mm at Pog (both *p* < 0.001); and vertical reduction, 0.99 mm at B and 1.32 mm at Pog (both *p* < 0.05).

**Table 4:** Changes in hard tissue and soft tissue landmarks on CBCT images

Landmarks	Δ*x* (T2–T1)			Δ*y* (T2–T1)			Δ*z* (T2–T1)		
	Mean	SD	*p* value	Mean	SD	*p* value	Mean	SD	*p* value
Hard tissue									
Ii	− 2.61	1.71	.000***	− 4.96	1.89	.000***	0.82	1.00	.040*
Lc dev	− 2.32	1.58	.000***	− 5.09	1.61	.000***	0.79	1.07	.026*
Lc ctl	− 2.72	1.89	.000***	− 6.18	1.70	.000***	0.98	1.35	.032*
Id	− 2.43	1.81	.000***	− 6.21	1.92	.000***	1.04	1.61	.043*
B	− 2.41	1.79	.000***	− 5.80	2.01	.000***	0.99	1.25	.030*
Pog	− 2.49	2.20	.003**	− 5.69	2.72	.000***	1.32	1.57	.019*
Me	− 2.42	2.18	.004**	− 5.81	3.05	.000***	1.20	1.45	.021*
Mf dev	− 1.95	1.57	.001**	− 4.98	2.42	.000***	0.31	0.81	.268
Mf ctl	− 1.82	1.67	.003**	− 6.41	2.29	.000***	0.72	0.72	.027*
Soft tissue									
Nose-related									
Al dev	− 0.09	0.21	.301	− 0.62	1.40	.184	0.08	0.32	.524
Al ctl	0.02	0.41	.930	− 0.25	0.91	.297	0.07	0.27	.532
Pn	− 0.04	0.18	.589	0.03	0.28	.906	− 0.02	0.22	.738
Sn	− 0.38	0.41	.106	− 0.09	0.73	.702	0.04	1.01	.889
Lip-related									
A’	− 0.50	0.80	.056	− 0.18	0.99	.538	0.34	1.55	.488
Ls	− 0.80	1.13	.047*	− 1.09	0.81	.028*	0.07	0.79	.755
Stms	− 0.81	1.16	.042*	− 1.12	1.10	.014*	0.81	1.53	.058
Ch dev	− 2.22	1.87	.008**	− 1.92	2.03	.027*	0.32	1.03	.506
Ch ctl	− 0.95	1.01	.031*	− 2.89	1.86	.011**	0.64	1.51	.179
Stmi	− 0.84	1.18	.036*	− 4.09	1.60	.000***	0.88	1.33	.125
Li	− 0.99	1.21	.036*	− 4.24	1.72	.000***	0.81	1.24	.066
Chin-related									
B’	− 1.18	1.27	.033*	− 4.24	2.08	.000***	0.94	1.31	.043*
Pog’	− 1.59	1.30	.015*	− 4.37	2.43	.000***	1.21	1.45	.019*
Me’	− 1.79	1.31	.009**	− 2.94	2.76	.003**	1.09	1.35	.030*
Cheek-related									
Zy dev	− 0.17	0.32	.443	0.21	1.22	.598	0.11	0.34	.291
Zy ctl	− 0.09	0.52	.430	− 0.23	1.06	.469	− 0.02	0.37	.925
Ck dev	− 0.20	1.28	.593	− 1.18	1.71	.041*	0.11	1.20	.797
Ck ctl	0.16	1.24	.575	− 0.81	2.37	.335	0.61	1.67	.316

Δ*x*, Δ*y*, and Δ*z* means changes in transverse axis, anteroposterior axis, and vertical axis, respectively

Paired *t* test was performed. SD indicates standard deviation; *p < 0.05; **p < 0.01; ***p < 0.001

### Changes in soft tissue landmarks and measurement

**Fig. 5:**
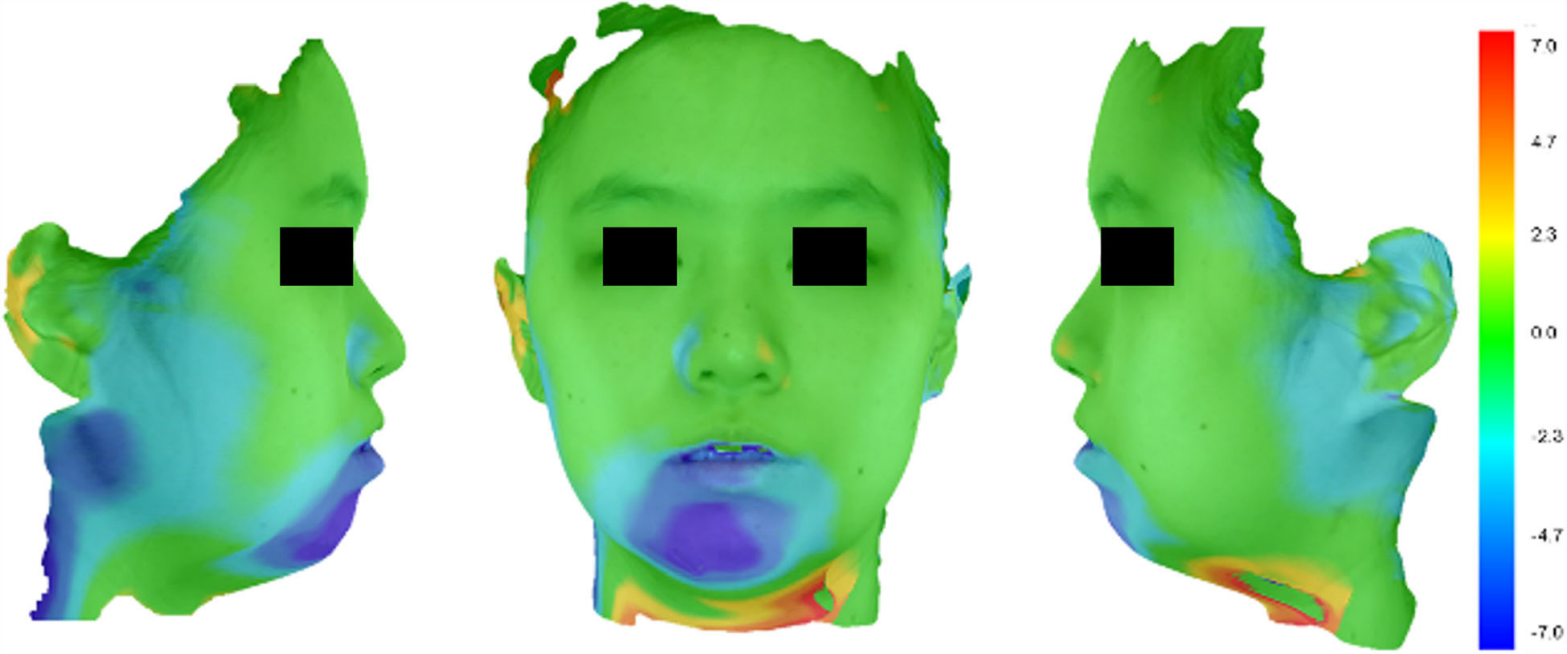
Superimposed color map image using concentration gradient of colors, before and after mandibular setback surgery. Increases in blue color gradient indicate greater inward displacement after MSS. Green color indicates no change. The greatest change occurred in the chin area with an increasing gradient from the lower lip to chin and from side to midline

Overall changes in the soft tissue after MSS were illustrated by superimposition of the facial scans (Fig. 5). Table 4 shows the changes in soft tissue landmarks after MSS in transverse, anteroposterior, and vertical axis on facial scan images.

In the transverse axis, there were significant changes in most landmarks related to the lips (Ls, Stms, Ch ctl, Stmi, Li, *p* < 0.05; Ch dev, *p* < 0.01, Table 4) and all landmarks related to the chin (B’, Pog’, Me’, all *p* < 0.05). Among the landmarks related to the nose and cheek, no landmarks showed statistically significant changes (*p* > 0.05).

In the anteroposterior direction, most landmarks of the lip and chin moved backward significantly (Ls, Stms, Ch dev, *p* < 0.05; Ch ctl, Me’, *p* < 0.01; Li, Stmi, B’, and Pog’, *p* < 0.001), except for A’ (*p* > 0.05). Landmarks of the nose and cheek did not exhibit any significant changes, except for Ck dev (*p* < 0.05).

In the vertical axis, significant upward movement was observed in the landmarks related to the chin (B’, Pog’, and Me’, *p* < 0.5), but not in the nose, lip, and cheek.

There was a significant decrease in lower facial height (− 2.17 mm, *p* < 0.01) and in lip width (1.97 mm, *p* < 0.05, Table 5).

**Table 5:** Linear changes in soft tissue measurements after mandibular setback surgery

Variables	Before surgery		After surgery		*p* value	ΔT2–T1	
	Mean	SD	Mean	SD		Mean	SD
Nasal width (Al dev–Al ctl)	39.65	3.04	39.60	3.01	.875	− 0.05	0.53
Lip width (Ch dev–Ch ctl)	47.72	3.37	45.75	4.02	.045*	− 1.97	1.74
Lower facial height (Sn–Me’)	68.23	5.45	66.06	4.88	.008**	− 2.17	1.15
Upper lip height (Sn–Stms)	19.79	2.87	18.81	2.30	.078	− 0.98	1.63
Lower lip height (Stmi–Pog’)	30.40	2.83	29.52	2.81	.153	− 0.88	1.91
Upper vermilion height (Ls–Stms)	5.58	1.40	5.57	1.39	.119	− 0.01	1.10
Lower vermilion height (Li–stmi)	9.49	1.70	9.16	1.48	.279	− 0.33	1.18

Paired *t* test was performed

*p < 0.05; **p < 0.01

### Correlations between changes in the hard and soft tissues

In the transverse axis, soft tissue landmarks related to the lip (A’, Ls, Ch dev, Ch ctl, Stmi, and Li) and chin (B’, Pog’, and Me’) showed significant correlations with hard tissue landmarks (Table 6). Correlations between corresponding hard and soft tissue landmarks were observed in the lower lip and chin (Li/Ii, *p* < 0.05; B’/B, *p* < 0.01; Pog’/Pog and Me’/Me, *p* < 0.001, Table 6). However, correlations between hard and soft tissues in nose- and cheek-related landmarks were not significant.

**Table 6:** Correlation coefficients (*p*) and ratio of soft-to-hard tissue movement (S/H) in the transverse direction

Landmarks		Ii		Lc dev		Lc ctl		Id		B		Pog		Me		Mf dev		Mf ctl	
		*p* value	(ratio)	*p* value	(ratio)	*p* value	(ratio)	*p* value	(ratio)	*p* value	(ratio)	*p* value	(ratio)	*p* value	(ratio)	*p* value	(ratio)	*p* value	(ratio)
Nose-related	Al dev	.320	(.034)	.369	(.049)	.268	(.033)	.363	(.037)	.365	(.037)	.427	(.036)	.436	(.037)	.305	(.046)	.251	(.049)
	Al ctl	− .219	(− .008)	− .159	(− .011)	− .135	(− .007)	− .301	(− .008)	− .284	(− .008)	− .284	(− .008)	− .246	(− .008)	− .212	(− .010)	− .151	(− .011)
	Pn	.049	(.015)	.196	(.022)	.008	(.015)	.155	(.016)	.310	(.017)	.264	(.016)	.253	(.017)	.242	(.021)	.168	(.022)
	Sn	.383	(.146)	.378	(.209)	.260	(.140)	.388	(.156)	.324	(.158)	.430	(.153)	.427	(.157)	.202	(.195)	.169	(.209)
Lip-related	A’	.603*	(.192)	.510	(.275)	.455	(.184)	.571	(.206)	.470	(.207)	.530	(.201)	.532	(.207)	.350	(.256)	.343	(.275)
	Ls	.718**	(.307)	.580	(.440)	.612*	(.294)	.705*	(.329)	.630*	(.332)	.701*	(.321)	.703*	(.331)	.520	(.410)	.503	(.440)
	Stms	.500	(.310)	.470	(.445)	.455	(.298)	.479	(.333)	.431	(.336)	.542	(.325)	.540	(.335)	.292	(.415)	.265	(.445)
	Ch dev	.395	(.851)	.585*	(1.220)	.361	(.816)	.347	(.914)	.219	(.921)	.340	(.892)	.349	(.917)	.389	(1.138)	.194	(1.220)
	Ch ctl	.288	(.364)	.302	(.522)	.590*	(.349)	.265	(.391)	.390	(.394)	.418	(.382)	.422	(.393)	.360	(.487)	.366	(.522)
	Stmi	.660*	(.322)	.570	(.462)	.651*	(.309)	.662*	(.346)	.586*	(.349)	.604*	(.337)	.601*	(.347)	.485	(.431)	.509	(.462)
	Li	.650*	(.379)	.602*	(.544)	.626*	(.364)	.641*	(.407)	.608*	(.411)	.695*	(.398)	.697*	(.409)	.490	(.508)	.476	(.544)
Chin-related	B′	.685*	(.452)	.606*	(.648)	.590*	(.434)	.680*	(.486)	.729**	(.490)	.727**	(.474)	.735**	(.488)	.540	(.605)	.538	(.648)
	Pog’	.788**	(.609)	.700*	(.874)	.610*	(.585)	.790**	(.654)	.776**	(.660)	.878***	(.639)	.846***	(.657)	.665*	(.815)	.628*	(.874)
	Me’	.765**	(.686)	.597*	(.984)	.544*	(.658)	.777**	(.737)	.742**	(.743)	.833***	(.719)	.850***	(.740)	.595*	(.918)	.583*	(.984)
Cheek-related	Zy dev	.389	(.065)	.353	(.093)	.278	(.063)	.394	(.070)	.310	(.071)	.403	(.068)	.399	(.070)	.232	(.087)	.195	(.093)
	Zy ctl	.062	(.034)	.145	(.049)	.216	(.033)	.118	(.037)	.101	(.037)	.078	(.036)	.069	(.037)	.189	(.046)	.216	(.049)
	Ck dev	.458	(.077)	.279	(.110)	.265	(.074)	.418	(.082)	.329	(.083)	.359	(.080)	.375	(.083)	.348	(.103)	.344	(.110)
	Ck ctl	− .329	(− .061)	− .192	(− .088)	− .203	(− .059)	− .295	(− .066)	− .173	(− .066)	− .206	(− .064)	− .246	(− .066)	− .260	(− .082)	− .313	(− .088)

Ratio means amount of change in the soft tissue/amount of change in the hard tissue; positive (+) value, the change with the same direction; negative (−) value, the opposite direction

Pearson correlation analysis was done

*p < 0.05; **p < 0.01; ***p < 0.001

In the anteroposterior axis, some soft tissue landmarks related to the lower lip (Stms, Stmi, and Li) and chin (B’ and Pog’) demonstrated a significant correlation with hard tissue landmarks (Table 7). Correlations between soft tissue and underlying corresponding hard tissue were observed (Li/Ii, B’/B, Pog’/Pog; *p* < 0.01). Correlations between hard and soft tissues in nose- and cheek-related landmarks were not significant except Pn and Lc ctl.

**Table 7:** Correlation coefficients (*p*) and ratio of soft-to-hard tissue movement (S/H) in the anteroposterior direction

Landmarks		Ii		Lc dev		Lc ctl		Id		B		Pog		Me		Mf dev		Mf ctl	
		*p* value	(ratio)	*p* value	(ratio)	*p* value	(ratio)	*p* value	(ratio)	*p* value	(ratio)	*p* value	(ratio)	*p* value	(ratio)	*p* value	(ratio)	*p* value	(ratio)
Nose-related	Al dev	− .121	(.125)	− .129	(.122)	− .405	(.100)	− .289	(.100)	− .216	(.107)	.147	(.109)	− .013	(.107)	− .101	(.124)	− .336	(.097)
	Al ctl	.011	(.050)	.098	(.049)	.110	(.040)	.216	(.040)	.214	(.043)	.310	(.044)	.451	(.043)	.152	(.050)	.196	(.039)
	Pn	.304	(− .006)	.520	(− .006)	.616*	(− .005)	.550	(− .005)	.549	(− .005)	.531	(− .005)	.508	(− .005)	.440	(− .006)	.547	(− .005)
	Sn	− .229	(.018)	− .009	(.018)	− .035	(.015)	− .129	(.014)	− .082	(.016)	.038	(.016)	.145	(.015)	− .171	(.018)	− .146	(.014)
Lip-related	A’	− .475	(.036)	.026	(.035)	.119	(.029)	− .008	(.029)	− .021	(.031)	.105	(.032)	.227	(.031)	− .098	(.036)	− .073	(.028)
	Ls	.042	(.079)	.196	(.077)	.242	(.063)	.119	(.063)	.108	(.067)	.135	(.069)	.315	(.067)	− .060	(.078)	.087	(.061)
	Stms	.696*	(.206)	.665*	(.200)	.510	(.165)	.500	(.164)	.590*	(.176)	.591*	(.179)	.390	(.176)	.628**	(.205)	.389	(.159)
	Ch dev	.215	(.387)	.591*	(.377)	.320	(.311)	.357	(.309)	.386	(.331)	.512	(.337)	.563	(.330)	.415	(.386)	.212	(.300)
	Ch ctl	.165	(.583)	.397	(.568)	.596*	(.468)	.422	(.465)	.363	(.498)	.380	(.508)	.417	(.497)	.155	(.580)	.387	(.451)
	Stmi	.598*	(.825)	.590*	(.804)	.675*	(.662)	.617 *	(.659)	.555	(.705)	.413	(.719)	.547	(.704)	.290	(.821)	.601*	(.638)
	Li	.760**	(.855)	.641*	(.833)	.600*	(.686)	.605 *	(.683)	.550	(.731)	.512	(.745)	.325	(.730)	.492	(.851)	.530	(.661)
Chin-related	B’	.789**	(.855)	.739*	(.833)	.725**	(.686)	.823***	(.683)	.822**	(.731)	.748**	(.745)	.560	(.730)	.738**	(.851)	.721**	(.661)
	Pog’	.731**	(.881)	.699*	(.859)	.622*	(.707)	.730**	(.704)	.769**	(.753)	.771**	(.768)	.644*	(.752)	.692**	(.878)	.620*	(.682)
	Me’	.323	(.593)	.287	(.578)	.150	(.476)	.221	(.473)	.258	(.507)	.413	(.517)	.405	(.506)	.220	(.590)	.125	(.459)
Cheek-related	Zy dev	.168	(− .042)	− .017	(− .041)	− .305	(−.034)	− .158	(− .034)	− .081	(− .036)	.199	(− .037)	.175	(− .036)	.014	(− .042)	− .209	(− .033)
	Zy ctl	.444	(.046)	− .079	(.045)	− .050	(.037)	.088	(.037)	.110	(.040)	.010	(.040)	.156	(.040)	.079	(.046)	.184	(.036)
	Ck dev	.150	(.238)	.383	(.232)	.121	(.191)	.030	(.190)	.042	(.203)	.201	(.207)	.093	(.203)	.090	(.237)	− .143	(.184)
	Ck ctl	.165	(.163)	− .039	(.159)	− .165	(.131)	− .173	(.130)	− .158	(.140)	− .180	(.142)	− .011	(.139)	− .137	(.163)	− .180	(.126)

Ratio means amount of change in the soft tissue/amount of change in the hard tissue; positive (+) value, the change with the same direction; negative (−) value, the opposite direction

Pearson correlation analysis was done

*p < 0.05; **p < 0.01; ***p < 0.001

In the vertical axis, only the soft tissue landmarks related to the chin (B’, Pog’, and Me’) showed significant correlations with hard tissue landmarks (Pog or Me)(Table 8).

**Table 8:** Correlation coefficients (*p*) and ratio of soft-to-hard tissue movement (S/H) in the vertical direction

Landmarks		Ii		Lc dev		Lc ctl		Id		B		Pog		Me		Mf dev		Mf ctl	
		*p* value	(ratio)	*p* value	(ratio)	*p* value	(ratio)	*p* value	(ratio)	*p* value	(ratio)	*p* value	(ratio)	*p* value	(ratio)	*p* value	(ratio)	*p* value	(ratio)
Nose-related	Al dev	− .310	(.098)	.288	(.101)	.177	(.082)	− .309	(.077)	− .283	(.081)	− .361	(.061)	− .266	(.067)	.242	(.258)	− .076	(.111)
	Al ctl	− .325	(.085)	− .065	(.089)	.020	(.071)	.085	(.067)	− .106	(.071)	− .031	(.053)	.068	(.058)	− .200	(.226)	.057	(.097)
	Pn	.029	(− .024)	− .322	(− .025)	− .269	(− .020)	.049	(− .019)	.126	(− .020)	.127	(− .015)	.250	(− .017)	.158	(− .065)	− .184	(− .028)
	Sn	− .209	(.049)	.133	(.051)	.108	(.041)	− .020	(.038)	.028	(.040)	.049	(.030)	.131	(.033)	− .064	(.129)	.033	(.056)
Lip-related	A’	− .201	(.415)	− .040	(.430)	− .018	(.347)	.116	(.327)	.198	(.343)	.147	(.258)	.341	(.283)	.121	(1.097)	.232	(.472)
	Ls	− .279	(.085)	.058	(.089)	− .064	(.071)	.085	(.067)	− .137	(.071)	.180	(.053)	.062	(.058)	− .449	(.226)	− .500	(.097)
	Stms	.067	(.988)	.219	(1.025)	.231	(.827)	− .036	(.779)	.205	(.818)	.020	(.614)	.213	(.675)	− .114	(2.613)	.016	(1.125)
	Ch dev	− .429	(.390)	− .142	(.405)	− .147	(.327)	− .329	(.308)	− .444	(.323)	− .306	(.242)	− .382	(.267)	− .214	(1.032)	.525	(.444)
	Ch ctl	− .129	(.780)	.170	(.810)	.109	(.653)	− .311	(.615)	− .289	(.646)	− .403	(.485)	− .329	(.533)	− .151	(2.065)	− .399	(.889)
	Stmi	− .020	(1.073)	.028	(1.114)	.340	(.898)	− .168	(.846)	− .193	(.889)	.231	(.667)	− .088	(.733)	− .403	(2.839)	− .070	(1.222)
	Li	.031	(.988)	.208	(1.025)	.350	(.827)	.052	(.779)	− .190	(.818)	.259	(.614)	− .060	(.675)	.528	(2.613)	− .195	(1.125)
Chin-related	B’	.055	(1.146)	.231	(1.190)	.340	(.959)	.223	(.904)	.326	(.949)	.584*	(.712)	.368	(.783)	− .418	(3.032)	− .069	(1.306)
	Pog’	.068	(1.476)	− .017	(1.532)	.020	(1.235)	.065	(1.163)	.215	(1.222)	.587*	(.917)	.310	(1.008)	− .115	(3.903)	− .375	(1.681)
	Me’	.222	(1.329)	− .198	(1.380)	− .038	(1.112)	.161	(1.048)	.505	(1.101)	.596*	(.826)	.628*	(.908)	.072	(3.516)	.219	(1.514)
Cheek-related	Zy dev	− .026	(.134)	.410	(.139)	.264	(.112)	.421	(.106)	.082	(.111)	− .025	(.083)	.132	(.092)	.275	(.355)	.060	(.153)
	Zy ctl	− .241	(− .024)	− .055	(− .025)	− .028	(− .020)	.016	(− .019)	− .250	(− .020)	− .224	(− .015)	− .170	(− .017)	.106	(− .065)	− .269	(− .028)
	Ck dev	− .320	(.134)	− .135	(.139)	− .034	(.112)	− .251	(.106)	−.305	(.111)	− .189	(.083)	− .200	(.092)	− .068	(.355)	− .402	(.153)
	Ck ctl	− .389	(.744)	.025	(.772)	− .071	(.622)	− .409	(.587)	− .435	(.616)	− .502	(.462)	− .468	(.508)	− .099	(1.968)	− .464	(.847)

Ratio means amount of change in the soft tissue/amount of change in the hard tissue; positive (+) value, the change with the same direction; negative (−) value, the opposite direction

Pearson correlation analysis was done

*p < 0.05; **p < 0.01; ***p < 0.001

There was a significant decrease in lower facial height (− 2.17 mm, *p* < 0.01), but not in the nasal width and measurements related to lip height.

### Ratios of soft tissue changes relative to hard tissue movement

In the transverse axis, there was an increasing gradient of ratios from Li/Ii to Me’/Me (Li/Ii, 0.38; B’/B, 0.49; Pog’/Pog, 0.64; and Me’/Me, 0.74, Table 6). In the anteroposterior axis, Li/Ii showed the greatest ratio (0.85, Table 7), followed by Pog’/Pog (0.77) and B’/B (0.73). In the vertical axis, Li/Ii, B’/B, Pog’/Pog, and Me’/Me were all larger than 0.9 (Li/Ii, 0.99; B’/B, 0.95; Pog’/Pog, 0.92; and Me’/Me, 0.91; Table 8).

## Discussion

This study used CBCT and facial scans to evaluate the changes and correlations in hard and soft tissue after MSS. This method has the advantage of transverse change measurement, as well as in the anteroposterior and vertical dimensions, by using an identical 3D coordinate system, compared with previous studies using a 2D lateral cephalogram with 3D facial scanning [4–6]. However, there are some potential errors in the study using CBCT and facial scans: inaccuracy in superimposing 3D facial scan on CBCT, setting the face into the coordinate system, and setting the reference landmarks.

The accuracy of superimposing the surface image of CBCT and the facial scan has been evaluated in previous studies, which reported that the image fusion was acceptable with a minimum error less than 1 mm [11–13]. In this study, to arrange the 3D image data of CBCT and surface scanning in an identical 3D coordinate system, image fusion was performed automatically using the Morpheus 3D software. According to Nelson et al., the Morpheus 3D software automatically registers the 3D facial scan with CBCT skin image to obtain optimal registration parameters. However, they also mentioned errors can occur when registering. They recommended taking CBCT and 3D facial scan with the patient in the same posture and minimizing the time lapse between CBCT and 3D facial scan [11].

Recently, some studies reported that the errors associated with setting reference landmarks on facial scan images were sub-millimeter and showed that facial soft tissue landmarks had moderate to high reliability and reproducibility [14, 15]. However, reproducibility can be decreased as an operator generates landmarks over 3D facial images. Therefore, to prevent potential errors, it is important to define landmark definitions clearly and skilled examiners should set the landmarks.

In this study, a distinctive pattern was observed in that the changes exhibited an increasing gradient to the chin in the transverse axis. But, Lim et al. reported landmarks of the lips did not show any significant transverse changes [6]. It might be due to selection criteria included in each study. Lim et al. included less than 3 mm of chin deviation and this study included below 4 mm of menton deviation. This study might include patients with more facial asymmetry and then more transverse correction of landmarks of the lips occurred after MSS [4].

The amount of changes increased from Stmi to Me’ (Table 4). Moreover, correlation of Me with the lower lip and chin increased from Stmi to Me’ (Table 6). There was an increasing gradient of ratios relative to Me from Li to Me’ (Table 6). Considering no change in the cheek, the influence of the muscle and soft tissue tension decreased as the distance from the area where the hard tissue changes increased [16].

Among the bilateral landmarks of the nose, lip, and chin, only Ch dev showed a significant change (*p* < 0.05, Table 4). This finding means that the Ch dev moved toward the midline, with greater movement compared with the corresponding Ch ctl. It might be due to asymmetric mandibular setback and consequent asymmetric changes in soft tissue tension. As a result, a decrease in lip width might occur (1.97 mm decrease, *p* < 0.05, Table 5). A significant decrease in lip width has been previously reported and explained by a decrease in soft tissue tension after MSS, because stretching of the soft tissue of the lower lip area in the prognathic mandible can be reduced after MSS [4].

In the anteroposterior direction, the ratio of backward movement was greater in the lower lip than in the chin, which was in accordance with the previous study using CT imaging [17]. These findings suggest that the lower lip could be under the influence of the muscle rather than the bone [17, 18], and this might be related to the inherent differences in the soft tissue between lip and chin.

Soft tissue changes in the upper lip (Ls, and Stms in the transverse axis; Stms in the anteroposterior axis; all *p* < 0.05, Table 4) seem to occur due to continuity of the orbicularis oris muscle and soft tissue tension, despite no movement of the maxilla [6, 17, 18]. Previous 2D cephalometric [16, 19] and 3D surface scan studies have reported backward movement of the upper lip [4–6]. However, the changes of the upper lip correlated less with the amount of mandibular setback than with the lower lip and chin (Tables 6 and 7), because the upper lip would be supported by the maxillary incisors rather than the mandible or lower incisors after MSS.

In the vertical axis, except some landmarks (B’, Pog’, Me’) related to the chin, there were few significant correlations between hard and soft tissue landmarks in the vertical axis (Table 8). The 1972 study by Worms, Speidel, and Isaacson reached similar conclusions despite the lack of sophisticated technology available today. These were in accordance with previous studies reporting that changes in the soft tissue did not closely follow those in hard tissue in the vertical plane compared with the anteroposterior and transverse planes [4, 17, 20]. These findings suggest that the vertical change of the soft tissue after surgery is hard to predict.

In relation to the vertical change of the landmarks, lower facial height was significantly decreased (*p* < 0.01, Table 5) due to upward and backward movement of the mandible and chin according to the inclination of the maxillary occlusal plane [4, 17]. However, there were no significant decreases in the heights of upper and lower lips or the vermilion, which is in accordance with previous findings [17]. Therefore, MSS can reduce the lower facial height rather than the lip height. This might be due to differences in the interlabial gap before and after surgery among cases.

This study showed the 3D soft tissue changes according to skeletal changes after mandibular setback surgery in Class III patients using the CBCT and facial scanning. The ratios of soft-to-hard tissue changes derived from this study would contribute to the database for planning prediction. As the techniques become more improved, it would be available for orthodontists to simulate the orthodontic treatment or orthognathic surgery, predict the treatment outcome more accurately, and set the better plan for soft tissue changes including facial appearance as well as hard tissue changes.

However, there may be a slight difference between the head posture and facial expression between CBCT and facial scanning due to taking time difference. Several studies are underway to overcome the technical limitations. Further clinical studies are expected for evaluating the 3D soft tissue changes of bimaxillary orthognathic surgery in facial asymmetry patients.

## Conclusions

Soft tissue changes after MSS correlated to underlying hard tissue movement in the transverse and anteroposterior aspects, but the correlation in the vertical aspect was uncertain. A distinct pattern of an increasing gradient from the upper and lower lips to the chin was observed in transverse changes.

## Data Availability

Data is included in the form of tables in the study.

## Abbreviations

2D: Two-dimensional
3D: Three-dimensional
CBCT: Cone-beam computed tomography
Ch: Cheilion
Ck: Cheek
ctl: Contralateral side
dev: Deviated side
FH: Frankfort horizontal
FZ: Frontozygomatic suture point
Ii: Incision inferior
Lc: Lower canine
Li: Labrale inferius
Ls: Labrale superius
Me: Menton
MSS: Mandibular setback surgery
N: Nasion point
Pn: Pronasale
Pog: Pogonion
Stmi: Stomion inferius
Stms: Stomion superius
