# Correction to: Three-dimensional soft tissue changes according to skeletal changes after mandibular setback surgery by using cone-beam computed tomography and a structured light scanner

**DOI:** 10.1186/s40510-019-0284-y

**Published:** 2019-07-26

**Authors:** Kyung-A Kim, Ye-Jin Chang, Su-Hyun Lee, Hyun-Joon An, Ki-Ho Park

**Affiliations:** 1 Department of Orthodontics, School of Dentistry, Kyung Hee University, 1 Hoegi-Dong, Dongdaemoon-Ku, Seoul 130-701 South Korea; 2 Department of Orthodontics, Graduate School, Kyung Hee University, Seoul, South Korea

**Correction to: Prog Orthod (2019) 20:25**

**https://doi.org/10.1186/s40510-019-0282-0**

In the publication of this article [1], there are errors in the Methods (Subjects) and Ethics approval and consent to participate section.

The error:

“This retrospective study was approved by the Institutional Review Board of XXXXX XXX University Medical Center (IRB No: K-2013-11018794). Skeletal Class III patients who had received bilateral sagittal split ramus osteotomy only by the same surgeon at the XXXXX XXX University Dental Hospital had been screened.”

Should instead read:

“This retrospective study was approved by the Institutional Review Board of Kyung Hee University Medical Center (IRB No: K-2013-11018794). Skeletal Class III patients who had received bilateral sagittal split ramus osteotomy only by the same surgeon at the Kyung Hee University Dental Hospital had been screened.”

The error:

“This retrospective study was approved by the Institutional Review Board of XXXXX XXX University Medical Center (IRB No: K-2013-11018794).”

Should instead read:

“This retrospective study was approved by the Institutional Review Board of Kyung Hee University Medical Center (IRB No: K-2013-11018794).”

This has now been updated in the original article [1].
